# Spiking Neural P Systems with Membrane Potentials, Inhibitory Rules, and Anti-Spikes

**DOI:** 10.3390/e24060834

**Published:** 2022-06-16

**Authors:** Yuping Liu, Yuzhen Zhao

**Affiliations:** Academy of Management Science, Business School, Shandong Normal University, Jinan 250014, China; 2021021027@stu.sdnu.edu.cn

**Keywords:** spiking neural P systems, universality, membrane potential, inhibitory rules, anti-spikes

## Abstract

Spiking neural P systems (SN P systems for short) realize the high abstraction and simulation of the working mechanism of the human brain, and adopts spikes for information encoding and processing, which are regarded as one of the third-generation neural network models. In the nervous system, the conduction of excitation depends on the presence of membrane potential (also known as the transmembrane potential difference), and the conduction of excitation on neurons is the conduction of action potentials. On the basis of the SN P systems with polarizations, in which the neuron-associated polarization is the trigger condition of the rule, the concept of neuronal membrane potential is introduced into systems. The obtained variant of the SN P system features charge accumulation and computation within neurons in quantity, as well as transmission between neurons. In addition, there are inhibitory synapses between neurons that inhibit excitatory transmission, and as such, synapses cause postsynaptic neurons to generate inhibitory postsynaptic potentials. Therefore, to make the model better fit the biological facts, inhibitory rules and anti-spikes are also adopted to obtain the spiking neural P systems with membrane potentials, inhibitory rules, and anti-spikes (referred to as the MPAIRSN P systems). The Turing universality of the MPAIRSN P systems as number generating and accepting devices is demonstrated. On the basis of the above working mechanism of the system, a small universal MPAIRSN P system with 95 neurons for computing functions is designed. The comparisons with other SN P models conclude that fewer neurons are required by the MPAIRSN P systems to realize universality.

## 1. Introduction

Artificial neural networks (ANNs) aim to empower artificial systems with information processing functions consistent with the complex yet efficient human brain system. They are progressively building models with greater functionality and a better fit with biological facts. The third-generation neural network models, spiking neural networks [1], adopt spikes for information encoding and transmission, which provides models with biological features consistent with realistic rationality. Spiking neural P systems (SN P systems) in the field of membrane computing has become a hotspot as one of the third-generation ANNs.

Membrane computing is a branch of natural computing [2]. The distributed parallel computing models obtained from the development of membrane computing are membrane systems, also known as P systems, and the study of P systems has been divided into three types according to the cell membrane structure or cell distribution: cell-like P systems, tissue-like P systems, and neural-like P systems. For the theoretical research of membrane computing, three types of P systems have been extended to obtain several universal computational models [3,4,5,6,7], and the computational complexity of extended P systems has been explored [8,9,10,11,12,13,14,15,16,17,18,19,20]. For the application research of membrane computing, existing studies have realized the integration of membrane computing with algorithms for applications in robot control [21,22], data modeling, and optimization [23,24,25,26], algorithms for solving NP problems [27,28,29], clustering algorithms [30,31,32,33,34,35,36,37], and image processing [38,39,40].

The spiking neural P system is an important component of the neural-like P system, an abstract simulation of the nervous system in which neurons communicate by sending spikes. Research on SN P systems has been highly dynamic in recent years, and existing research falls into two main areas: theoretical research and application research.

Regarding the theoretical research of SN P systems, there are two main parts including the proposal of variants and the evaluation of computational performance. Several variants of SN P systems have been obtained, mainly via the introduction of various biological mechanisms or features. The purpose is to bring the system more in line with biological principles and facts, as well as to improve computational performance, such as reducing resources without losing computational power. Existing studies abstract biological phenomena and facts, and they extend the systems via the continuous introduction of various biological mechanisms. The extension of the system objects has been achieved via the introduction of calcium-producing astrocytes [41] and the use of five types of spikes [42]. The extension of the system rules has been realized through the control of neuronal firing using a threshold mechanism [43,44], introducing spikes distribution mechanism in rules [45], applying white rules [46], and applying evolutionary rules and communication rules [47]. By introducing the structural plasticity mechanism [48,49], the original SN P systems have achieved a larger degree of extension. The extension of the system operation was achieved via the introduction of the extended channel rule [50], synaptic delay [51], dendritic and axonal computation [52], a generalized use of rules [53] and the application of four sequential working strategies in the system [54,55]. The various variants of the SN P systems obtained still possess computational generality.

Furthermore, theoretical research has focused on evaluating the computational performance of variants of SN P systems, mainly including the computational generality and the computational complexity of the system. The evaluation of the computational generality of the proposed system focuses on proving the Turing universality, also called generality, via the simulation of the computation of the universal register machines with different functions. The extended SN P systems obtained from existing studies have all been proved to be Turing universal as number generation and accepting devices [41,42,44,45,50,51,52]. The studies addressing the computational complexity of systems include temporal complexity and spatial complexity. The temporal complexity of the system computation is assessed using the ability of the proposed variant to solve the NP-hard problems in polynomial time [56,57]. The spatial complexity is assessed via the construction of a universal system with fewer computational resources, i.e., the neurons. For example, 109 neurons are required to construct a universal dynamic threshold SN P system [44], while a SN P system with target indications requires only 15 neurons to achieve generality [45].

Regarding the application research of SN P systems, studies have focused on combining SN P systems with algorithms to solve real-world problems, and then they have evaluated the performance of the proposed algorithm with the help of experimental results on data sets and comparisons with other algorithms. SN P systems and numerous universal variants have been realized for applications in different real-world domains, such as performing basic arithmetic operations [58], simulating Boolean circuits [59], solving classification problems [60], fault diagnosis [61,62], recognizing English letters [63], image processing [64,65,66], modeling [67], and time series prediction [68,69,70]. As one of the third-generation of ANNs, SN P systems are considered to have significant development potential.

Recently, Wu et al. [71] introduced the concept of polarization into SN P systems and obtained the spiking neural P systems with polarization (PSN P systems), which changed the previous control mechanism of neuronal firing in SN P systems. Instead of using regular expressions for the trigger conditions of rules, the neuron firing was controlled by judging the neuron-associated polarizations (+, 0, −) as the rule trigger conditions. The application of polarizations made the information exchange of neurons in the system more consistent with biological facts, and the systems were shown to still be Turing universal. Wu et al. [72] then introduced a new coding object, the anti-spikes, into the PSN P systems (PASN P systems), in order to provide better computational performance and further simplification. In addition, the computational universality of the PSN P system in asynchronous mode [73] and sequential mode [74,75] is demonstrated. Subsequently, Yang et al. [76] added the feature of multiple channels to the PSN P systems (SNP–MCP systems). By introducing the spiking rules on synapses, Jiang et al. [77] obtained a new variant (PSNRS P systems) requiring less computational resources to realize computational generality.

The new variant of the SN P systems constructed in this paper is motivated by the following two biological mechanisms.

**Figure 1:**
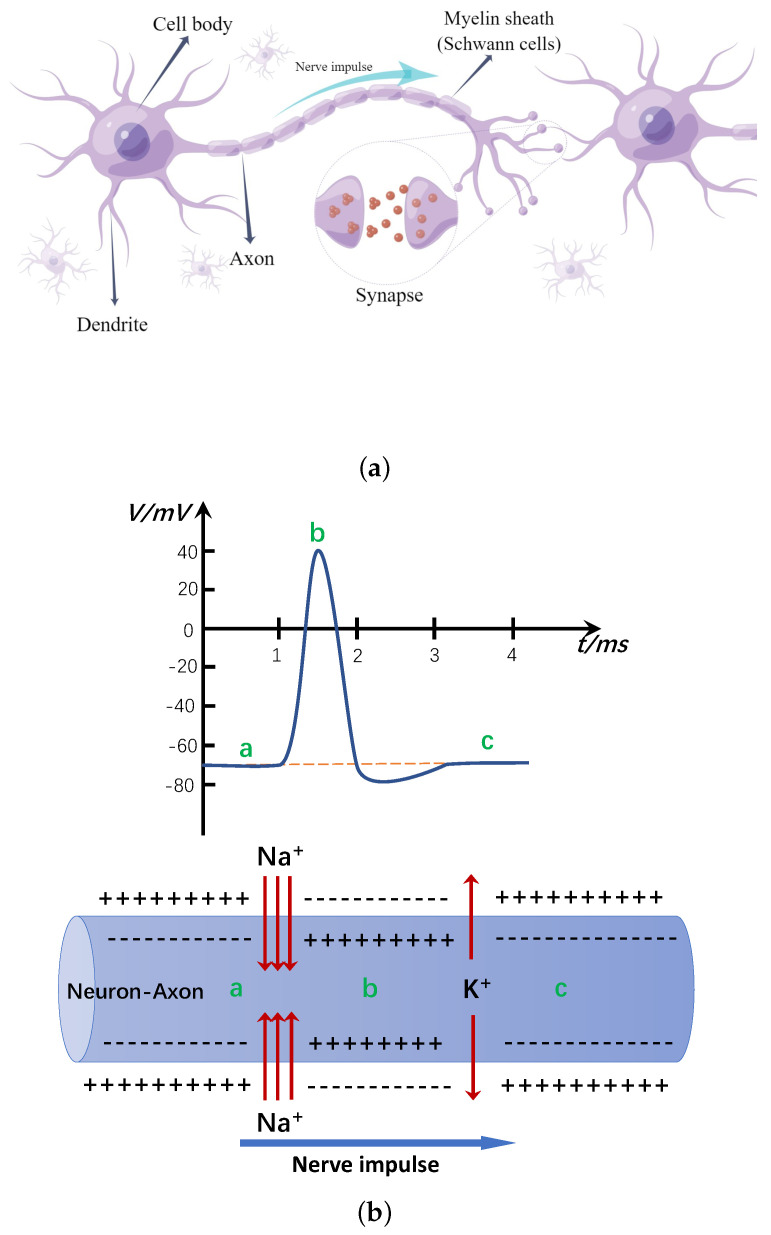
Conduction of the nerve impulse. (**a**) Schematic diagram of the basic structure of a neuron. (By Figdraw, www.figdraw.com accessed on 10 June 2022 ); (**b**) Schematic diagram of the spike voltage variation of the action potential with time.

- Neurons of the nervous system contain ions that carry a certain amount of charge (either positive or negative), and the presence of charge forms the transmembrane potential difference (also called potential) of the nerve cell. When a neuron receives a stimulus flow directionally, it forms an electric current, changes the transmembrane potential difference, generates an action potential, and counts to conduct this electrical signal along the cell membrane [78]. Thus, as shown in Figure 1, the conduction of excitation in the nervous system is the process of action potential conduction, and the phenomena of charge aggregation, flow, and transport exist in the cell membrane of neurons. Based on the PSN P systems, we introduce the concept of membrane potential according to the above biological phenomena, as a way to update the number of charges and polarization states of a neuron by considering the aggregation within the neuron and the charge transmission between neurons. Together with the polarization state and the number of charges, the membrane potential of a neuron is composed, and the membrane potential is used as the triggering condition of rules, which provides more powerful control over the systematic computation. The resulting model is constructed to better simulate the characteristics of neurons and the working mechanism of the nervous system.
- There are two main types of synapses between neurons according to their synaptic effects on neuronal activity: excitatory synapses and inhibitory synapses. The presence of excitatory synapses enables the transmission of information between neurons, and the operation mechanism of this synapse can be well modeled by the application of rules of consumption or transmission of spikes by systemic neurons. For inhibitory synapses, such synapses can cause postsynaptic neurons to generate inhibitory postsynaptic potentials, which in turn have an inhibitory effect on the excitation of neurons. Peng et al. [79] formalized the effect mechanism of inhibitory synapses as inhibitory rules within systematic neurons. The extension of the rule-triggering conditions not only made the firing behavior of neurons limited by the contained rules, but also controlled by the state of neurons connected to the current neuron through inhibitory synapses, effectively modeling the mechanism of action of inhibitory synapses in the nervous system.

Based on the above motivation, we also introduced the application of anti-spikes in the new systems to make the systems have better computational performance, and the obtained spiking neural P systems with membrane potentials, inhibitory rules, and anti-spikes (MPAIRSN P systems for short) are more in line with the biological mechanisms. In addition, the new inhibitory rules introduced in the systems consider the neuronal membrane potential as the rule triggering condition, and we update the form of the inhibitory rules as $\left(A_{en},\bar{A_{in(i,j)}}\right)/b^{c}\to b^{\prime };\beta \;$; the firing of the current neuron $\sigma_{i}$ needs to satisfy that the membrane potential $A_{i}$ is consistent with the membrane potential $A_{en}$ required by the rule, and that the membrane potential $A_{j}$ of the inhibitory neuron $\sigma_{j}$, which is connected to the current neuron $\sigma_{i}$ by inhibitory synapses, cannot belong to the set of membrane potential $A_{in(i,j)}$ required by the rule. That is, the condition $A_{i}=A_{en}\wedge A_{j}\notin A_{in(i,j)}$ needs to be satisfied for the current neuron to fire. The introduction of inhibitory rules achieves a more powerful control over the neurons in the system and it can further reduce the computational resources required by the universal MAIRSN P systems.

The research contribution of this paper is mainly focused on the following two points.

- We introduce the concept of membrane potential in the SN P systems and propose a new rule-triggering mechanism: using the membrane potential of a neuron as the condition. In addition, the inhibitory rule with membrane potential as the rule triggering condition is updated and applied, which in turn leads to the proposed MPAIRSN P systems.
- The proposed MPAIRSN P systems are shown as Turing universal operating in generating and accepting modes. A small universal MPAIRSN P system is constructed, using 95 neurons and allowing for the computation of functions. Compared with other variants of SN P systems adopting polarizations, the general MPAIRSN P systems require fewer computation resources and have faster computation speed.

For the remainder of this paper, Section 2 proposes the definition of the MPAIRSN P system and displays a small system as an introductory example of how the MPAIRSN P system operates, which has the capability of generating arbitrary nonzero natural numbers. Section 3 proves the Turing universality of the MPAIRSN P systems, mainly operating in generating and accepting modes, respectively. Section 4 constructs a small universal MPAIRSN P system containing 95 neurons for computing functions. Section 5 gives conclusions and the future research outlook.

## 2. SN P Systems with Membrane Potentials, Inhibitory Rules, and Anti-Spikes

In this section, a formal definition of the MPAIRSN P system is given to introduce the related concepts. Moreover, we design a small MPAIRSN P system as an example for illustrating the working mechanism. This small system implements the function of generating arbitrary nonzero natural numbers.

### 2.1. Definition

An MPAIRSN P system consisting of m≥1 neurons is represented as a tuple:

$$\Pi =(O,\sigma_{1},\cdots ,\sigma_{m},syn,syn^{\prime },in,out),$$

where:

(1) $O=\{a,\bar{a}\}$ is an alphabet consisting of two characters, where *a* and $\bar{a}$ denote a spike and an anti-spike, respectively.

(2) $\sigma_{1}$,$\sigma_{2}$,…,$\sigma_{m}$ are the *m* neurons contained in the system, and each neuron can be expressed as a tuple $\sigma_{i}=\left(A_{i},n_{i},R_{i}\right)$, 1≤i≤m, where:

(a) $A_{i}=\left(PAR_{i},X_{i}\right)$ is the initial membrane potential of neuron $\sigma_{i}$, which contains two main components, the polarization of the membrane potential $PAR_{i}\in \left\{+,0,-\right\}$, and the number of charges $X_{i}\in N$ contained in the neuron; *N* is the set of natural numbers.

(b) $n_{i}$ is the initial number of spikes/anti-spikes contained in the neuron. If $n_{i}\geq 0$, then the neuron $\sigma_{i}$ contains $n_{i}$ spikes; if $n_{i}<0$, then the neuron $\sigma_{i}$ contains $-n_{i}$ anti-spikes.

(c) $R_{i}$ is the set of rules contained in the neuron. The rules are mainly of the following types.

i. Standard rules: $A/b^{c}\to b^{\prime };\beta$,

where *A* is the firing condition, β∈{+,0,−}, $b,b^{\prime }\in O$, c≥1.

ii. Inhibitory rules: $\left(A_{en},\bar{IA_{in(i,j)}}\right)/b^{c}\to b^{\prime };\beta \;$,

where $A_{en}$ and $IA_{in(i,j)}$ together constitute the firing condition, β∈{+,0,−}, $b,b^{\prime }\in O$, c≥1.

iii. $a\bar{a}\to \lambda$ is called the annihilating rule. This rule enforces a higher priority than the standard rules and inhibitory rules above.

(3) $syn=\{\left.(i,j)\right|1\leq i\neq j\leq m\}$ is the set of standard synapses between neurons. $syn^{\prime }=\left\{\left.(i,j)\right|1\leq i\neq j\leq m\right\}$ is the set of inhibitory synapses between neurons, where *j* is the label of the inhibitory neuron and *i* is the label of the inhibited neuron.

(4) in,out∈{1,2,⋯,m} are used to distinguish the input and output neurons in the system, respectively. If the system does not contain the input neuron or output neuron, it is omitted.

For the operation of the MPAIRSN P system, since it is a requirement to ascertain neuronal polarization by calculating the number of contained charges, we give the following conventions of charge calculation.

(1) Multiple positive (or negative) charges are allowed to accumulate within a neuron.
(2) A positive charge and a negative can cancel each other out and disappear.
(3) Receiving any number of neutral charges does not change neuronal polarization state.

There are three main types of rules in the MPAIRSN P system: standard rules, inhibitory rules, and annihilating rules.

The standard rule takes the form of $A/b^{c}\to b^{\prime };\beta \;$. The triggering condition of this rule restricts the membrane potential of the neuron, i.e., both the polarization and the charge number are required to satisfy the condition. The firing rule is available to be applied when the membrane potential state of neuron $\sigma_{i}$ is such that $A_{i}=A$, and the number of spikes or anti-spikes contained in $\sigma_{i}$ is at least *c* so that sufficient spikes are available to achieve firing.

In particular, if the firing condition of the rule restricts only the polarization of the neuron, then the standard firing rule is simplified to the form $PAR/b^{c}\to b^{\prime };\beta$. The rule is applicable if the polarization state of neuron $\sigma_{i}$ satisfies $PAR_{i}=PAR$ and the number of spikes contained in $\sigma_{i}$ is at least *c*.

Moreover, if the standard rule is triggered without generating spikes or anti-spikes, i.e., $b^{\prime }=\lambda$, this rule is called the standard forgetting rule. In this case, only one charge β is produced and then transmitted.

In summary, if a neuron $\sigma_{i}$ satisfies the trigger condition of its standard firing rule, *c* spikes or anti-spikes are consumed, the neuron generates one spike or anti-spike and a charge β that are simultaneously sent to the successive neuron connected to $\sigma_{i}$ via synapse, updating the number of spikes and the membrane potential state of the successive neuron. If the rule triggered is a forgetting rule, only charge β is produced and then transmitted between neurons.

Similarly, standard forgetting rules of the form $A^{\prime }/b^{s}\to \lambda ;\beta^{\prime }\;$ and $PAR^{\prime }/b^{s}\to \lambda ;\beta^{\prime }\;$ consume *s* spikes or anti-spikes in the neuron, and generate no spikes or anti-spikes but only produce one charge, which is sent to subsequent neurons $\sigma_{j}$ connected to $\sigma_{i}$ via synapses. This type of rules can be applied as soon as the membrane potential state of neuron $\sigma_{i}$ coincides with the membrane potential requirement of the rule, i.e., $A_{i}=A^{\prime }$, or the polarization of neuron $\sigma_{i}$ satisfies $PAR_{i}=PAR^{\prime }$. Moreover, the number of spikes or anti-spikes in $\sigma_{i}$ must be greater than *s*.

**Figure 2:**
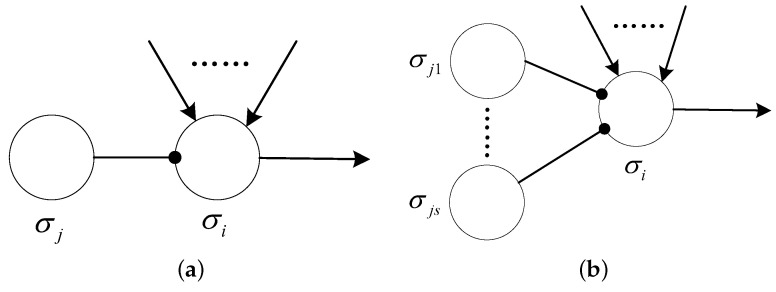
Rules. (**a**) Inhibitory rules: $\left(A_{en},\bar{IA_{in(i,j)}}\right)/b^{c}\to b^{\prime };\beta$. (**b**) Extend inhibitory rules: $\left(A_{en},\bar{IA_{in(i,js)}},\cdots ,\bar{IA_{in(i,js)}}\right)/b^{c}\to b^{\prime };\beta$.

The inhibitory rules take the form shown in Figure 2. For the formal representation of the rule, $A_{en}$ indicates the membrane potential condition that needs to be satisfied for the current neuron $\sigma_{i}$ to fire, and the subscript (i,j) of $IA_{in(i,j)}$ indicates the presence of a directed inhibitory synapse between the current neuron $\sigma_{i}$ and its inhibitory neuron $\sigma_{j}$.

The application of each inhibitory rule is controlled by the membrane potential $A_{i}$ or the polarization $PAR_{i}$ of the current neuron $\sigma_{i}$, and the membrane potential $A_{j}$ or the polarization $PAR_{j}$ of the inhibitory neuron $\sigma_{j}$ connected to neuron $\sigma_{i}$ via the inhibitory synapse. Taking the form of $\left(A_{en},\bar{A_{in(i,j)}}\right)/b^{c}\to b^{\prime };\beta$, the inhibitory rule is applicable to neuron $\sigma_{i}$ if its membrane potential $A_{i}$ coincides with the membrane potential condition of the rule $A_{en}$, i.e., $A_{i}=A_{en}$, with its inhibitory neuron $\sigma_{j}$ containing the membrane potential $A_{j}$ that does not belong to the set $IA_{in(i,j)}$, i.e., $A_{j}\notin IA_{in(i,j)}$. In other words, the triggering of the inhibitory rule requires that the expression $A_{i}=A_{en}\wedge A_{j}\notin IA_{in(i,j)}$ holds. Additionally, the number of spikes or anti-spikes contained in neuron $\sigma_{i}$ is at least *c*.

Similarly, the simplified inhibitory rule takes the form of $\left(PAR_{en},\bar{IPAR_{in(i,j)}}\right)/b^{c}\to b^{\prime };\beta$, which is available to neuron $\sigma_{i}$ if the polarization states of current neuron $\sigma_{i}$ and its inhibitory neuron $\sigma_{j}$ enable the expression $PAR_{i}=PAR_{en}\wedge PAR_{j}\notin IPAR_{in(i,j)}$, and the number of spikes or anti-spikes contained in neuron $\sigma_{i}$ is at least *c*. Analogously, this type of rules only restricts the polarizations of the current neuron $\sigma_{i}$ and its inhibitory neuron $\sigma_{j}$.

If triggered, the inhibitory firing rule consumes *c* spikes or anti-spikes in the neuron $\sigma_{i}$, generating one spike or anti-spikes and one charge β, which are sent to the successive neuron connected to $\sigma_{i}$ via synapse. For the inhibitory rule, if $b^{\prime }=\lambda$, it is an inhibitory forgetting rule. Similarly, the triggering of this rule does not generate spikes or anti-spikes, but only a charge.

It is worth noting that, unlike the standard form of synaptic connection, the inhibitory neuron is connected to the current neuron via the inhibitory synapse, also called the inhibitory arc. The inhibitory arc is represented in the directed graph as a directed arc with a solid circle, whereas the standard synapse is represented by a directed arc with an arrow. For the directed inhibitory arc in a graph, there are the following conventions.

(1) If there is an inhibitory arc between neurons $\sigma_{i}$ and $\sigma_{j}$, then there is no standard arc between them, i.e., there is no longer a standard synaptic connection between two neurons.
(2) If neuron $\sigma_{j}$ is an inhibitory neuron to neuron $\sigma_{i}$, there is no transmission of spikes, and charges between two neurons. Moreover, the application of the inhibitory rules in $\sigma_{i}$ performs no effect on its inhibitory neuron $\sigma_{j}$ in terms of changing the number of spikes and the membrane potential of neuron $\sigma_{j}$. The transmission of spikes, anti-spikes and charges does not take place in the inhibitory arc. Thus, inhibitory neuron $\sigma_{j}$ only functions as a control of neuron $\sigma_{i}$ in terms of its firing.

If a neuron is controlled by more than one inhibitory neuron, there are extended inhibitory rules that are applicable. As shown in Figure 2b, neuron $\sigma_{i}$ has multiple inhibitory neurons $\sigma_{j1},\cdots ,\sigma_{js}$. Then, for the extended inhibitory firing rule $(A_{en},\bar{IA_{in(i,js)}},\cdots ,$$\bar{IA_{in(i,js)}})/b^{c}\to b^{\prime };\beta$, the firing of the neuron $\sigma_{i}$ requires that the neuron $\sigma_{i}$ and its inhibitory neurons $\sigma_{j1},\cdots ,\sigma_{js}$ satisfy the condition that $A_{i}=A_{en}\wedge A_{j1}\notin IA_{in(i,j1)}\wedge \cdots \wedge A_{js}\notin IA_{in(i,js)}$.

The annihilation rule takes the form of $a\bar{a}\to \lambda$, i.e., one spike and one anti-spike cancel each other out when they meet in the same neuron. The execution of this rule is time independent and uncontrolled by the neuronal membrane potential or polarization. Therefore, the two types of spikes are unable to be present in the same neuron simultaneously.

Generally, any MPARISN P system containing *m* neurons can be represented by a tuple $\Pi =(O,\sigma_{1},\cdots ,\sigma_{m},syn,syn^{\prime },in,out)$, or by a topological directed graph. The directed graph representation can help us to visualize the structure and the operation process of the system. Then, in order to facilitate the explanation of the operation process, the MPAIRSN P systems in the latter part of this paper are mainly introduced with the help of directed graphs.

Topologically, an MPAIRSN P system Π can be expressed as a directed graph with inhibitory arcs, where *m* neurons are shown as *m* nodes containing initial spikes and rules, represented by rounded rectangles, and the synaptic connections between neurons are denoted as directed arcs, including the standard directed arcs and the newly introduced inhibitory arcs. For each neuron $\sigma_{i}$ in the system, we assign a label *i*, an initial number of spikes, and an initial membrane potential. The neuron label and its initial membrane potential are represented by the tuple $\left(i,\left(PAR_{i},X_{i}\right)\right)$ placed next to the rounded rectangle representing the neuron, where $PAR_{i}$ represents its polarization and $X_{i}$ is the corresponding number of charges. The initial spikes are then located in the first row inside the rounded rectangle, and if the neuron contains no initial spikes, only the rule is included.

As the state of neuron $\sigma_{i}$ in a system at a given instance is formulated by the number of spikes jointly with the membrane potential, it follows that the overall computational configuration of the system Π at the particular instance *t* is denoted as $C_{t}=<A_{1}/n_{1},A_{2}/n_{2},\cdots ,A_{m}/n_{m}>$, $n_{i}\in Z$, where $A_{i}$ is the membrane potential, $n_{i}$ is the number of spikes, and *Z* is the set of integers. The computation performed in the system is presented as a transformation of the systematic configuration, starting from the initial configuration $C_{0}$. The systematic neurons apply three types of rules to perform the computation, and the process is represented as $C_{0}\Rightarrow C_{1}\Rightarrow \cdots \Rightarrow C_{h}$, where $C_{h}$ is the terminate configuration. The computational process ends when no more rule is available to be applied in the system. Additionally, for any calculation, there is a corresponding spikes sequence encoded by 01 that reflects the behavior of the output neuron, where 0 signifies silence of the output neuron, while 1 indicates that the output neuron fires and transmits spikes.

The MPAIRSN P system Π is operable in both the generating mode and the accepting mode. When the system operates in the generation mode, it can serve as a number generation device and export computation results by the output neuron $\sigma_{out}$. The computation result can be defined as either the time interval between the first two spikes, or the total number of spikes sent by $\sigma_{out}$. We denote the computation results generated by the MPAIRSN P system Π as N(Π), or $N_{2}\left(\Pi \right)$ in case the computation result is defined in the former manner. When the system performs in accepting mode, an input neuron is introduced into the system, and the output neuron is removed. The number n is encoded as a spikes sequence consisting of 01, and then it is read by the input neuron $\sigma_{in}$. If the computation terminates, it indicates that the number n is accepted by the system, and the set of numbers accepted by the system Π is noted as $N_{acc}\left(\Pi \right)$.

### 2.2. An Example

**Figure 3:**
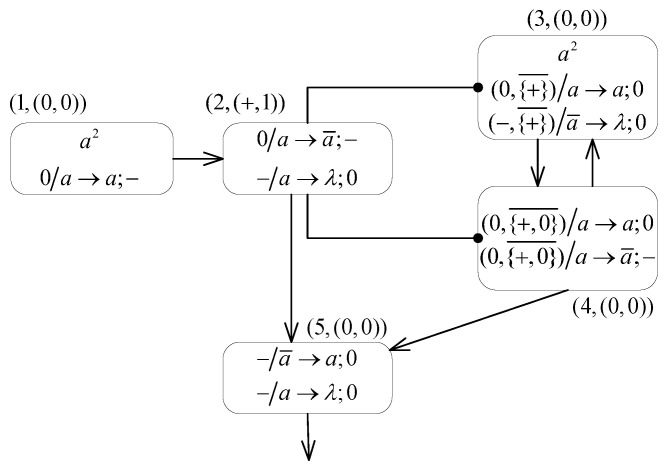
A small MPAIRSN P system $\Pi_{e}$ as an illustrative example.

An example is given in Figure 3 to explain the working mechanism of MPAIRSN P systems, which is a small system $\Pi_{e}$ capable of generating arbitrary positive natural numbers n(n>0). The MPAIRSN P system $\Pi_{e}$ containing five neurons is represented as a tuple:

$$\Pi_{e}=\left(O,\sigma_{1},\sigma_{2},\cdots ,\sigma_{5},syn,syn^{\prime },in,out\right),$$

where:

$O=\{a,\bar{a}\}$,
$\sigma_{1}=\left(\left(0,0\right),2,\left\{0/a\to a;-\;\right\}\right)$,
$\sigma_{2}=\left(\left(+,1\right),0,\left\{0/a\to \bar{a};-\;,-/a\to \lambda ;0\;\right\}\right)$,
$\sigma_{3}=\left(\left(0,0\right),2,\left\{\left(0,\bar{\left\{+\right\}}\right)/a\to a;0\;,(-,\bar{\left\{+\right\}}/\bar{a}\to \lambda ;0\;\right\}\right)$,
$\sigma_{4}=\left(\left(0,0\right),0,\left\{\left(0,\bar{\left\{+,0\right\}}\right)/a\to a;0\;,\left(0,\bar{\left\{+,0\right\}}\right)/a\to \bar{a};-\;\right\}\right)$,
$\sigma_{5}=\left(\left(0,0\right),0,\left\{-/\bar{a}\to a;0\;,-/a\to \lambda ;0\;\right\}\right)$,
syn={(1,2),(3,4),(4,3),(2,5),(4,5)},
$syn^{\prime }=\left\{\left(3,2\right),\left(4,2\right)\right\}$, and
$out=\sigma_{5}$.

For ease of understanding and interpretation, we also represent the system $\Pi_{e}$ as a directed graph (Figure 3), where both neuron $\sigma_{1}$ and neuron $\sigma_{3}$ contain two initial spikes, and neuron $\sigma_{2}$ carries the initial membrane potential (+,1). There are, in total, five neurons in the system, where neuron $\sigma_{5}$ is the output neuron. Each neuron is assigned a tuple $\left(i,\left(PAR_{i},X_{i}\right)\right)$. For example, for the tuple (2,(+,1)) assigned to neuron $\sigma_{2}$, 2 is the label of the neuron, and the initial membrane potential is (+,1), i.e., representing that the neuron $\sigma_{2}$ carries the positive polarization and contains one positive charge. Moreover, the neuron $\sigma_{2}$ is connected to neuron $\sigma_{3}$ and neuron $\sigma_{4}$ via two inhibitory synapses, meaning that the firing of neuron $\sigma_{3}$ and neuron $\sigma_{4}$ is controlled by their neuron $\sigma_{2}$.

The initial configuration of the system in this example is $C_{0}=<\left(0,0\right)/2,\left(+,1\right)/0,$ (0,0)/2,(0,0)/0,(0,0)/0>. The calculation result of this system is defined as the interval between the first two spikes received in the environment.

At the initial step, since neuron $\sigma_{1}$ with neutral polarization contains two spikes, the rule 0/a→a;− is applied to send one spike and one negative charge to neuron $\sigma_{2}$. Meanwhile, neuron $\sigma_{3}$ which remains of neutral polarization and contains two spikes, is unable to apply the inhibitory firing rule as $PAR_{2}\in \left\{+\right\}$ without satisfying the firing condition. Thereby, the system configuration of step 1 can be obtained as $C_{1}=<\left(0,0\right)/1,\left(0,0\right)/1,\left(0,0\right)/2,\left(0,0\right)/0,\left(0,0\right)/0>$. At step 1, as the neuron $\sigma_{1}$ contains one spike and remains neutral in polarization, rule 0/a→a;− continues to be applied to send another spike and one negative charge to neuron $\sigma_{2}$. Additionally, neuron $\sigma_{2}$ with neutral polarization contains one spike, where its rule $0/a\to \bar{a};-\;$ can be triggered to send one spike and one negative charge to neuron $\sigma_{5}$. Neuron $\sigma_{3}$ satisfies the trigger condition $PAR_{3}=0\wedge PAR_{2}\notin \left\{+\right\}$ of the rule $\left(0,\bar{\left\{+\right\}}\right)/a\to a;0$, and it sends one spike to neuron $\sigma_{4}$ by the application of this inhibitory firing rule. We then have $C_{2}=<\left(0,0\right)/0,\left(-,1\right)/1,\left(0,0\right)/1,\left(0,0\right)/1,\left(-,1\right)/\left(-1\right)>$ at step 2. At this step, it becomes empty after neuron $\sigma_{2}$ fires without producing spikes and charges, since the neuron $\sigma_{2}$ satisfies the trigger condition of the forgetting rule −/a→λ;0. Neuron $\sigma_{5}$ with negative polarization contains an anti-spike, allowing the application of the rule to send the first spike to the environment. Additionally, the application of the rule $\left(0,\bar{\left\{+\right\}}\right)/a\to a;0\;$ continues in neuron $\sigma_{3}$, sending one spike to neuron $\sigma_{4}$. The polarization of neuron $\sigma_{4}$ and the polarization of its inhibitory neuron $\sigma_{2}$ fulfill $PAR_{4}=0\wedge PAR_{2}\notin \left\{+,0\right\}$, and then both rule $\left(0,\bar{\left\{+,0\right\}}\right)/a\to a;0\;$ and rule $\left(0,\bar{\left\{+,0\right\}}\right)/a\to \bar{a};-\;$ are applicable. The uncertainty selection of rules applied in neuron $\sigma_{4}$ can be performed, and then the following two cases exist.

Case 1: Assume that the rule $\left(0,\bar{\left\{+,0\right\}}\right)/a\to \bar{a};-\;$ is applied in neuron $\sigma_{4}$ at step 2, and an anti-spike and a negative charge are sent to neuron $\sigma_{3}$ and neuron $\sigma_{5}$, consuming one spike while still receiving a spike from neuron $\sigma_{3}$, then neuron $\sigma_{4}$ contains one spike and has neutral polarization. Therefore, both neuron $\sigma_{3}$ and neuron $\sigma_{5}$ contain an anti-spike and have negative polarization. Then, there is $C_{3}=<\left(0,0\right)/0,\left(-,1\right)/0,\left(-,1\right)/\left(-1\right),\left(0,0\right)/1,$ (−,2)/(−1)> at step 3. Subsequently, the second spike is sent to the environment from neuron $\sigma_{5}$ by applying rule $-/\bar{a}\to a;0\;$ at step 4. Once no rule can be applied in the system, i.e., the computation halts, the time interval between the first spike received in the environment is 1, i.e., the natural number 1 computed by the system is obtained.

Case 2: Suppose that by applying the rule $\left(0,\bar{\left\{+,0\right\}}\right)/a\to a;0\;$ at step 2, neuron $\sigma_{4}$ sends one spike to neuron $\sigma_{3}$ and neuron $\sigma_{5}$. After consuming one spike while receiving one spike from neuron $\sigma_{3}$, there is one spike contained in neuron $\sigma_{4}$ with positive polarization. Then, there is $C_{3}=<\left(0,0\right)/0,\left(-,1\right)/0,\left(0,0\right)/1,\left(0,0\right)/1,\left(-,1\right)/1>$. The application of rule $\left(0,\bar{\left\{+\right\}}\right)/a\to a;0\;$ continues in neuron $\sigma_{3}$ at step 3, sending one spike to neuron $\sigma_{4}$, and neuron $\sigma_{5}$ applies the forgetting rule −/a→λ;0 without producing spikes. Since neuron $\sigma_{4}$ contains one spike and carries neutral polarization, the uncertain selection of rule $\left(0,\bar{\left\{+,0\right\}}\right)/a\to a;0\;$ or $\left(0,\bar{\left\{+,0\right\}}\right)/a\to \bar{a};-\;$ for application can be performed again. In this case, we assume that neuron $\sigma_{4}$ consistently applies rule $\left(0,\bar{\left\{+,0\right\}}\right)/a\to a;0\;$ selectively at each step until step k(k>4). Then, we have $C_{i}=<\left(0,0\right)/0,\left(-,1\right)/0,\left(0,0\right)/1,\left(0,0\right)/1,\left(-,1\right)/1>$, (4≤i≤k). The rule $\left(0,\bar{\left\{+,0\right\}}\right)/a\to \bar{a};-\;$ is selected to be applied in neuron $\sigma_{4}$ at step *k*, and then an anti-spike and a negative charge are generated and sent to neuron $\sigma_{3}$ and neuron $\sigma_{5}$. At moment k+2, neuron $\sigma_{5}$ contains one spike for consumption of the rule $-/\bar{a}\to a;0\;$ to send the second spike to the environment. Then, the time interval between the first two spikes received in the environment until the calculation stops is k−3(k>4), giving an arbitrary natural number that is greater than 1.

As mentioned above, the small MPAIRSN P system in this example allows for the generation of arbitrary nonzero natural numbers. The set of positive natural numbers generated by this system is denoted as $N_{2}\left(\Pi_{e}\right)=\left\{n|n\geq 1\right\}$.

## 3. The Computational Universality of MPAIRSN P Systems

Through the design of the MPAIRSN P systems to simulate the register machine in accepting and generating modes, respectively, this section explores the computational universality of the MPAIRSN P system to demonstrate that all recursive enumerated sets of numbers can be generated or accepted by the system.

A register machine is represented as a tuple: $M=(m,H,l_{0},l_{h},I)$, where *m* is the number of registers, *H* is the set of labels corresponding to instructions, while $l_{0},l_{h}\in H$ are the starting and halting instruction labels, respectively. I is the set of instructions distinguished by labels in H, and two types of instructions are included: $l_{i}:\left(ADD\left(r\right),l_{j},l_{k}\right)$ and $l_{i}:\left(SUB\left(r\right),l_{j},l_{k}\right)$. Typically, the family NRE is used to characterize the set of numbers that can be generated or accepted by the register machine. The computational results of the MPAIRSN P system containing m neurons are denoted by $N_{\alpha }PAIRSNP_{m}^{n}$, α∈{2,acc}, with at most *n* rules within each neuron.

### 3.1. The MPAIRSN P System as a Number Generating Device

The register machine *M* working in generating mode is available to serve as a number generator. As for its configuration, at the initial state, each register in *M* is empty, and the machine starts working by executing the instruction $l_{0}$. Then, the machine calculates by executing a series of ADD and SUB instructions. Eventually, the machine’s computation is terminated by executing the instruction $l_{h}$, at which point the number n stored in register 1 is the number generated by *M*.

**Theorem** **1.**
$N_{2}SNP_{*}^{2}\left(ch_{3}\right)=NRE.$

**Proof.**  According to the Turing–Church thesis, the relation $N_{2}SNP_{*}^{2}\left(ch_{3}\right)\subseteq NRE$ holds [80]. It remains only to prove that $NRE\subseteq N_{2}SNP_{*}^{2}\left(ch_{3}\right)$. Therefore, we mainly consider the proof using the MPAIRSN P system $\Pi_{1}$ to simulate the computation of the register machine *M* working in the generative mode. We assume that for the configuration of the machine at a given step, all its registers are empty and that the value stored in register 1 of them does not decrease during the computation.

**Figure 4:**
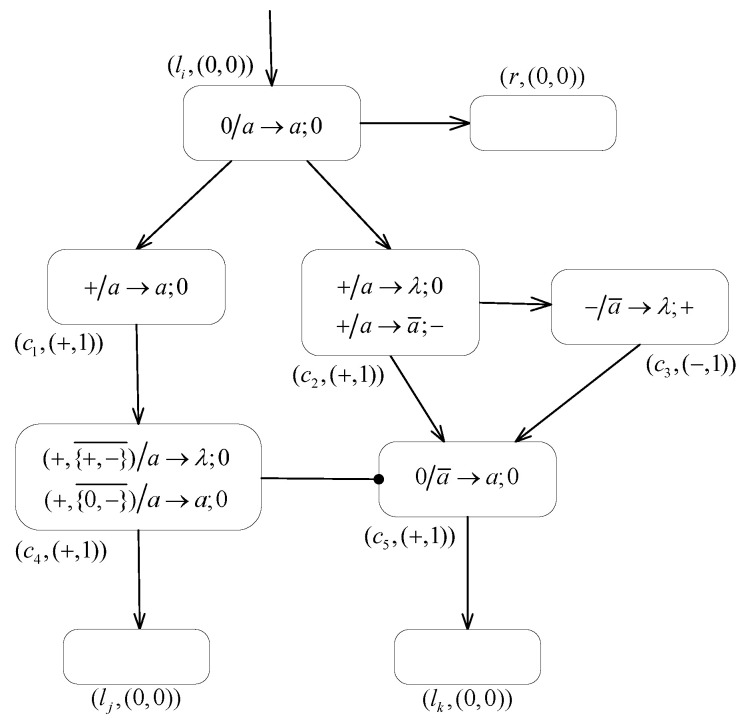
The ADD module of system $\Pi_{1}$.

**Figure 5:**
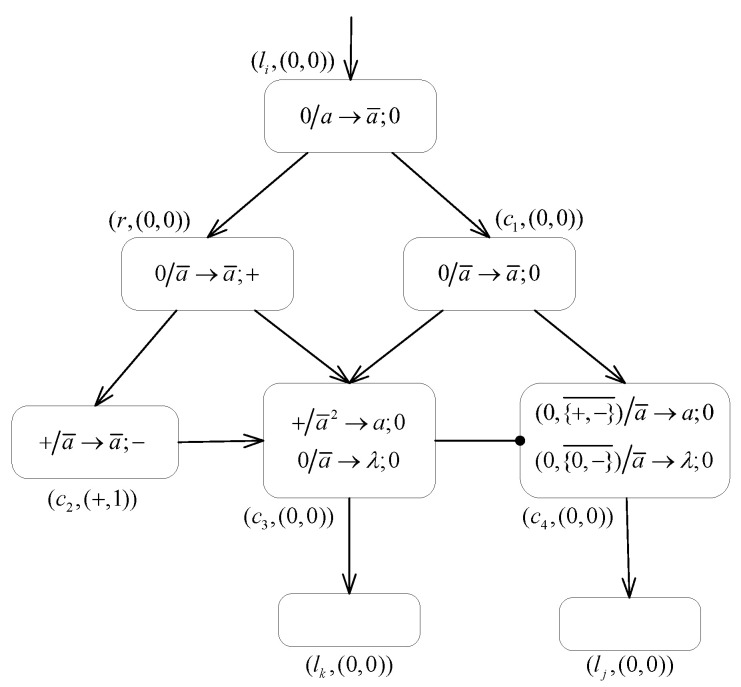
The SUB module of system $\Pi_{1}$.

**Figure 6:**
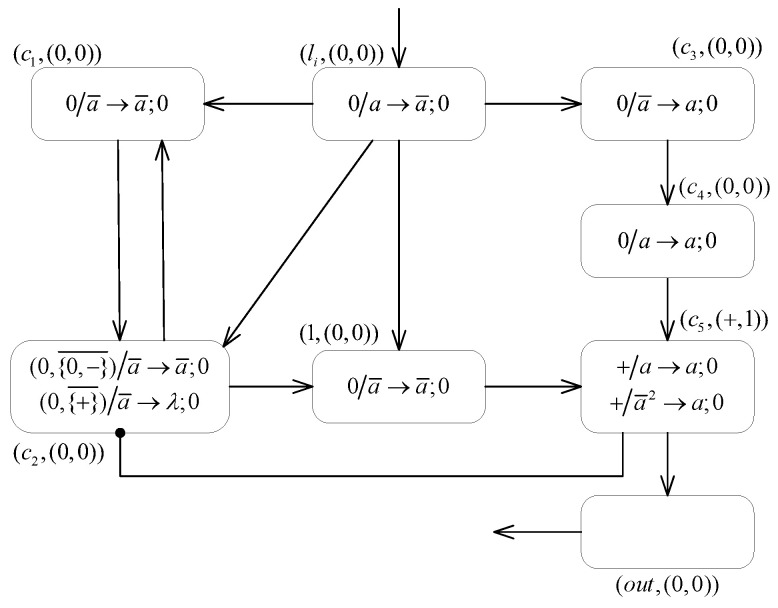
The FIN module of system $\Pi_{1}$.

In the following contents, an MPAIRSN P system $\Pi_{1}$ is designed to simulate the register machine $M_{1}$ working in generation mode, which can act as a number generation device (or a number generator). The system $\Pi_{1}$ is designed with three types of modules containing the ADD module, the SUB module, and the FIN module, as shown in Figure 4, Figure 5 and Figure 6, respectively.

In order to associate with the register machine $M_{1}$, we set the neuron $\sigma_{r}$ in system $\Pi_{1}$ to correspond to a register *r* in machine $M_{1}$, and the number of spikes contained in the neuron $\sigma_{r}$ is equal to the value stored in the corresponding register *r*. The neuron $\sigma_{l_{i}}$ in system $\Pi_{1}$ corresponds to instruction $l_{i}$ in the machine. In addition, the auxiliary neurons $\sigma_{c_{i}}\left(i=1,2,\cdots \right)$ of the module associated with a neuron $\sigma_{l_{i}}$ are added, and they are uniquely related.

In the initial state, each neuron in the system has an initial membrane potential; the neuron $\sigma_{l_{0}}$ receives a spike for triggering the system computation, and the rest of the neurons are empty. During the computation steps, neuron $\sigma_{l_{i}}$ with a neutral polarization fires as soon as it receives a spike, i.e., the system begins to simulate instruction $l_{i}:\left(OP\left(r\right),l_{j},l_{k}\right)\left(OP=ADD,SUB\right)$, which triggers the work of the relevant module. Following that, neuron $\sigma_{l_{j}}$ or neuron $\sigma_{l_{k}}$ receives one spike and starts to simulate instruction $l_{j}$ or $l_{k}$, which triggers the calculation of the corresponding module. If neuron $\sigma_{l_{h}}$ receives one spike and fires, then system $\Pi_{1}$ successfully simulates the computation of the register machine $M_{1}$ in generative mode, and the computation result is output by the FIN module where neuron $\sigma_{l_{h}}$ is located. We define the time interval $t_{2}-t_{1}$ between the first two spikes output by the neuron $\sigma_{out}$ to the environment as the computation result, and the value corresponds to the number stored in register 1.

The procedure and the details of the proofs for the simulations of the ADD, SUB, and FIN modules of the system $\Pi_{1}$ are given below.

**(1) The ADD module (Figure 4) simulates an ADD instruction $l_{i}:\left(ADD\left(r\right),l_{j},l_{k}\right)$.**

Suppose that at step *t*, an ADD instruction $l_{i}$ is triggered and neuron $\sigma_{l_{i}}$ picks up one spike available for firing. Then, at step t+1, $\sigma_{c_{1}}$, $\sigma_{c_{2}}$ and $\sigma_{r}$ all contain one spike derived from the firing of neuron $\sigma_{l_{i}}$. Following this, neuron $\sigma_{c_{1}}$ fires by applying the rule +/a→a;0, while one of rules +/a→λ;0 and $+/a\to \bar{a};-\;$ can be applied indeterministically in neuron $\sigma_{c_{2}}$. The following two cases are possible.

- If the forgetting rule +/a→λ;0 is applied, then there is one spike in neuron $\sigma_{c_{4}}$ with positive polarization, as well as its inhibitory neuron $\sigma_{c_{5}}$ also carries positive polarization. Then, the triggering condition of the inhibitory spiking rule $\left(+,\bar{\left\{0,-\right\}}\right)/a\to a;0\;$ is satisfied, i.e., $PAR_{4}=+\wedge PAR_{2}\notin \left\{0,-\right\}$. As soon as neuron $\sigma_{l_{j}}$ receives one spike from neuron $\sigma_{c_{4}}$, this simulated computational procedure activates the module associated with instruction $l_{j}$.
- If the rule applied in neuron $\sigma_{c_{2}}$ is $+/a\to \bar{a};-$, then at step t+2, there is an anti-spike contained in neuron $\sigma_{c_{5}}$ with neutral polarization. That is, rule $0/\bar{a}\to a;0\;$ is applied inside neuron $\sigma_{c_{5}}$ and one spike generated is sent to neuron $\sigma_{l_{k}}$. Under such a case, neuron $\sigma_{c_{4}}$ is reset to empty by applying the inhibitory forgetting rule $\left(+,\bar{\left\{+,-\right\}}\right)/a\to a;0$. The neuron $\sigma_{c_{5}}$ then receives one positive charge generated by the application of the forgetting rule $-/\bar{a}\to \lambda ;+\;$ in neuron $\sigma_{c_{3}}$ to restore its initial polarized state. Thus, this simulation computation activated the module associated with instruction $l_{k}$.

In summary, the ADD module successfully simulates the execution of the ADD instruction $l_{i}:\left(ADD\left(r\right),l_{j},l_{k}\right)$. The acceptance of one spike by neuron $\sigma_{l_{i}}$ starts the computation of the ADD module, followed by the implementation of adding one to the number of spikes contained in the neuron $\sigma_{r}$, corresponding to adding one to the value in the register r in the machine. Sending one spike to neuron $\sigma_{l_{j}}$ or $\sigma_{l_{k}}$ by indeterminacy corresponds to the indeterminate execution of instruction $l_{j}$ or $l_{k}$.

**(2) The SUB module (Figure 5) simulates a SUB instruction $l_{i}:\left(SUB\left(r\right),l_{j},l_{k}\right)$.**

Suppose that at step *t*, neuron $\sigma_{l_{i}}$ receives one spike and fires. Following that, as neuron $\sigma_{c_{1}}$ fires, both neurons $\sigma_{c_{3}}$ and $\sigma_{c_{4}}$ contain one anti-spike from neuron at step t+2. Again, depending on the value stored in register *r*, corresponding to changes occurring in neuron $\sigma_{r}$ and the operation of the module, the following two cases exist.

- If the value in register *r* is 0, and accordingly, the neuron $\sigma_{r}$ is empty, then at step t+1, neuron $\sigma_{r}$ consumes one anti-spike using the rule $0/\bar{a}\to \bar{a};+\;$ to transmit one positive charge and one anti-spike to neurons $\sigma_{c_{2}}$ and $\sigma_{c_{3}}$. At moment t+2, neuron $\sigma_{c_{3}}$ has two unconsumed anti-spikes with positive polarization, satisfying the firing condition of rule $+/{\bar{a}}^{2}\to a;0$, enabling one spike to be sent to neuron $\sigma_{l_{k}}$. In addition, the neuron $\sigma_{c_{4}}$, which contains one anti-spike with neutral polarization, has the triggering condition of its inhibitory forgetting rule $\left(0,\bar{\left\{0,-\right\}}\right)/\bar{a}\to \lambda ;0\;$ satisfied, i.e., $PAR_{c_{4}}=0\wedge PAR_{c_{3}}\notin \left\{0,-\right\}$, and then it becomes empty without generating spikes or charges upon the application of the rule. At this point, neuron $\sigma_{c_{2}}$ also fires with the application of the rule $+/\bar{a}\to \bar{a};-$. At the next step, neuron $\sigma_{c_{3}}$ receives one negative charge from neuron $\sigma_{c_{2}}$ and becomes neutral, which means that it reverts to its initial polarization. The received anti-spike is then consumed by the application of the forgetting rule $0/\bar{a}\to \lambda ;0$, and neuron $\sigma_{c_{3}}$ eventually becomes empty. In general, this process activates the computational module associated with the instruction $l_{k}$.
- If the value in register *r* is n≥1 and correspondingly neuron $\sigma_{r}$ contains *n* spikes, then at step t+1, after receiving an anti-spike from neuron $\sigma_{l_{i}}$, neuron $\sigma_{r}$ consumes one spike by the application of the annihilation rule $a\bar{a}\to \lambda$, and the number of spikes it contains becomes n−1. At step t+2, neuron $\sigma_{c_{3}}$ only receives one anti-spike from neuron $\sigma_{c_{3}}$ and maintains neutral polarization, and then it becomes empty after applying the forgetting rule $0/\bar{a}\to \lambda ;0$. In contrast, the neuron $\sigma_{c_{4}}$, which contains one anti-spike and carries neutral polarization, satisfies simultaneously with its inhibitory neuron the trigger condition $PAR_{c_{4}}=0\wedge PAR_{c_{3}}\notin \left\{+,-\right\}$ of the inhibitory spiking rule $\left(0,\bar{\left\{+,-\right\}}\right)/\bar{a}\to a;0$, so that one spike can be sent to neuron $\sigma_{l_{j}}$. Then, the process activates the computational module associated with the instruction $l_{j}$.

In summary, the SUB module successfully simulates the SUB instruction $l_{i}:\left(SUB\left(r\right),l_{j},l_{k}\right)$. Similarly, starting from the acceptance of one spike by neuron $\sigma_{l_{i}}$, the instruction $l_{j}$ or $l_{k}$ successively is simulated depending on the number of spikes (n=0 or n≥1) contained in neuron $\sigma_{r}$, respectively.

Remarkably, during the simulated computation of systematic instructions $l_{i}:\left(SUB\left(r\right),l_{j},l_{k}\right)$ by system $\Pi_{1}$, there is an unavoidable situation that multiple instructions act on the same register according to the different instruction labels, so that mutual interference exists in the computation between the SUB modules. Specifically, for the simulated instruction $l_{i}:\left(SUB\left(r\right),l_{j},l_{k}\right)$ of the SUB module shown in Figure 4, there may be another instruction $l_{s}$ acting on the register *r* as well. Then, during the simulation of instruction $l_{i}$, for the case where the value stored in register *r* is 0, the corresponding neuron $\sigma_{r}$ sends both an anti-spike and a positive charge to auxiliary neurons $\sigma_{c_{2}}$ and $\sigma_{c_{3}}$ in the SUB module associated with instruction $l_{s}$. Thus neuron $\sigma_{c_{3}}$ contains one anti-spike and carries positive polarization, which is not enough to apply the spiking rule $+/{\bar{a}}^{2}\to a;0$. As for neuron $\sigma_{c_{2}}$, it can apply rule $+/\bar{a}\to \bar{a};-\;$ to send an anti-spike and a negative charge to neuron $\sigma_{c_{3}}$. Subsequently, neuron $\sigma_{c_{3}}$ contains two anti-spikes with neutral polarization, turning empty after applying the standard forgetting rule $0/\bar{a}\to \lambda ;0$. As a result, the auxiliary neurons $\sigma_{c_{2}}$ and $\sigma_{c_{3}}$ in the SUB module associated with instruction $l_{s}$ affected by the firing of neuron $\sigma_{r}$ can be restored to their initial state. In summary, the interference between the SUB modules does not affect the correctness of the computation process.

**(3) The FIN module (Figure 6) outputs the computation result.**

If the computation process operates up to the instruction $l_{h}$, the computation is finished, and the result is output by the FIN module. At step *t*, neuron $\sigma_{l_{h}}$ receives one spike, and the FIN module is activated. At step t+1, neuron $\sigma_{c_{3}}$ receives the anti-spikes from neuron $\sigma_{l_{h}}$, and the execution of the rule $0/\bar{a}\to a;0\;$ generates one spike. Neurons $\sigma_{c_{4}}$, $\sigma_{c_{5}}$, and the output neuron $\sigma_{out}$ fire in the subsequent step. At step t+4, the output neuron $\sigma_{out}$ sends its first generating spike into the environment. In addition, starting from step t+1, neuron $\sigma_{c_{1}}$ consumes one anti-spike for firing, neuron $\sigma_{c_{2}}$ and its inhibitory neuron $\sigma_{c_{5}}$ also satisfy the triggering condition of the inhibitory spiking rule $\left(0,\bar{\left\{0,-\right\}}\right)/\bar{a}\to \bar{a};0\;$, i.e., $PAR_{c_{2}}=0\wedge PAR_{c_{5}}\notin \left\{0,-\right\}$. Then neurons $\sigma_{c_{1}}$ and $\sigma_{c_{2}}$ begin the process of exchanging one anti-spike with each other, and they continuously fire. Additionally, at each subsequent step, neuron $\sigma_{c_{2}}$ sends one anti-spike to neuron $\sigma_{1}$, gradually annihilating the *n* spikes contained inside it.

Until step t+n, neuron $\sigma_{1}$ becomes empty, but continues to receive anti-spikes from neuron $\sigma_{c_{2}}$. Then, at step t+n+1, the neuron $\sigma_{1}$ is able to trigger the rule $0/\bar{a}\to \bar{a};0$, and sends one anti-spike to neuron $\sigma_{c_{5}}$. The excitation condition of the inhibitory spiking rule in neuron $\sigma_{c_{2}}$ is then not satisfied at step t+n+2, as the polarization of neuron $\sigma_{c_{5}}$ changes to neutral. However, the inhibitory forgetting rule $\left(0,\bar{\left\{+\right\}}\right)/\bar{a}\to \lambda ;0\;$ is eventually triggered, leaving the neuron $\sigma_{c_{2}}$ empty. At step t+n+3, the neuron $\sigma_{c_{5}}$ contains a total of two anti-spikes and carries negative polarization, allowing the rule $-/{\bar{a}}^{2}\to a;0\;$ to be applied. Thus, the second spike is sent to the environment by the neuron $\sigma_{out}$ at step t+n+4. The calculation result output by this module is (t+n+4)−(t+4)=n, which corresponds to the value in register 1.

In summary, the MPAIRSN P system $\Pi_{1}$ correctly simulates the register machine $M_{1}$ working in the generating mode, which applies three types of polarizations, with, at most, two rules per neuron in the system. Therefore, Theorem 1 holds. □

The proposal of the MPAIRSN P system is an improvement of the spiking neural P system with polarization, by introducing membrane potentials to complete the application of polarization in the SN P system, which makes the model more consistent with biological facts. Therefore, we compare the required resources (Table 1) of the four SN P systems, using polarizations as number generating devices. The comparison data are taken from the references. For the computation resources, the comparisons are the maximum number of rules contained in each neuron, and the number of auxiliary neurons required for each instruction module.

In terms of the number of neurons needed in each module, the MPAIRSN P system and the SNP–MCP system use a fewer number of neurons compared to the remaining three SN P systems with polarizations. The FIN module, which is used to output the result, is applied only once at the end of the computation process. Then, it is mainly the number of neurons in the ADD and SUB modules that affect the size of the computation resources. From the average value of the auxiliary neurons in the two modules, the MPAIRSN P systems require fewer computational resources.

### 3.2. The MPAIRSN P System as a Number Accepting Device

The register machine *M*, operating in accepting mode, serves to receive the natural number *n*, with all registers contained being empty in the initial state. The machine introduces the number *n* to be analyzed from the environment, stores the value *n* in the first register, and then starts the computational work from the execution instruction $l_{0}$. If the computation processing of the machine reaches the termination instruction $l_{h}$, the number *n* is considered to be received by the machine $M_{2}$. The set of numbers that can be received by the machine *M* is recorded as $N_{acc}\left(M\right)$.

**Theorem** **2.**
$N_{2}SNP_{*}^{2}\left(ch_{3}\right)=NRE.$

**Proof.**  As is similar to the proof of Theorem 1, we only need to prove that the expression $N_{acc}SNP_{*}^{2}\subseteq NRE$ holds. To simulate the register machine $M_{2}=\left(m,H,l_{0},l_{h},I\right)$ operating in accepting mode, we design the MPAIRSN P system $\Pi_{2}$, which serves as a number accepting device. The system $\Pi_{2}$ mainly consists of three types of computing modules: the deterministic ADD module, the SUB module, and the INPUT module.

**Figure 7:**
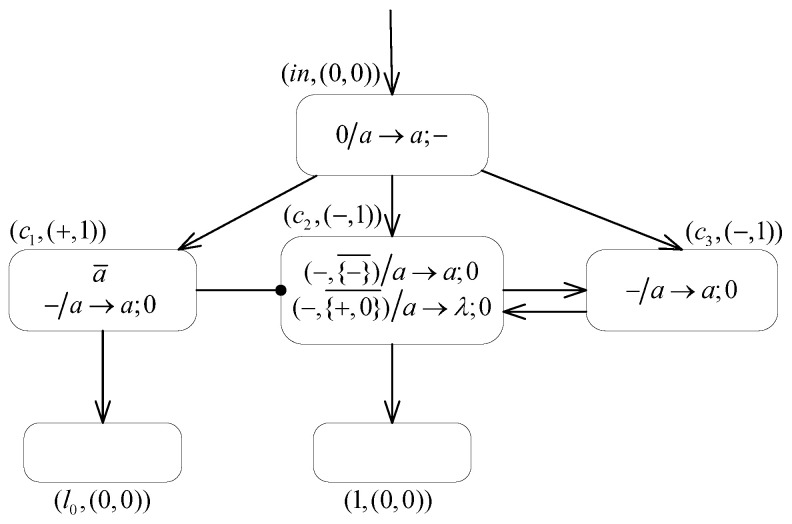
The INPUT module of system $\Pi_{2}$.

**(1) The INPUT module (Figure 7) introduces the number n into the system.**

Suppose that at step *t*, the spikes sequence $10^{n-1}1$ is introduced into the INPUT module of system $\Pi_{2}$. Then, the neuron $\sigma_{in}$ receives the first spike from the environment and applies the rule 0/a→a;− to fire. After receiving a negative charge from neuron $\sigma_{in}$, neuron $\sigma_{c_{1}}$ takes the neutral polarization. The membrane potentials of neurons $\sigma_{c_{2}}$ and $\sigma_{c_{3}}$ both become −2, where one spike is contained in every neuron. Then, during step t+2 and subsequent computation steps, since the formula $PAR_{c_{2}}=-\wedge PAR_{c_{1}}\notin \left\{-\right\}$ always holds, neuron $\sigma_{c_{2}}$ can continuously apply the inhibitory spiking rule $\left(-,\bar{\left\{-\right\}}\right)/a\to a;0\;$ to fire and transmit one spike to the neurons $\sigma_{c_{2}}$ and $\sigma_{1}$. At the same time, neuron $\sigma_{c_{3}}$ also applies the rule −/a→a;0 to continuously supply spikes to neuron $\sigma_{c_{2}}$. Thus, there is a constant exchange of spikes between neurons $\sigma_{c_{2}}$ and $\sigma_{c_{3}}$, and each step adds one to the number of spikes contained in neuron $\sigma_{1}$.

At step t+n, neuron $\sigma_{in}$ receives the second spike from the spikes sequence, and the rule 0/a→a;− is applied again to generate one negative charge and one spike. Then at step t+n+1, neuron $\sigma_{c_{1}}$ contains one spike and carries the negative polarization. The rule −/a→a;0 is triggered, which sends a generated spike to neuron $\sigma_{l_{0}}$. However, neuron $\sigma_{c_{2}}$ is no longer able to apply the inhibitory spiking rule, but satisfies $PAR_{c_{2}}=-\wedge PAR_{c_{1}}\notin \left\{+,0\right\}$. The inhibitory forgetting rule $\left(-,\bar{\left\{+,0\right\}}\right)/a\to \lambda ;0\;$ is triggered and leaves the neuron $\sigma_{c_{2}}$ eventually empty. At this point, there are, in total, *n* spikes stored in neuron $\sigma_{1}$. At the step t+n+2, neuron $\sigma_{l_{0}}$ receives one spike, which excites the module associated with instruction $l_{0}$ and thus starts the analog computation, while the number *n* is introduced into the neuron $\sigma_{1}$ in the system.

**Figure 8:**
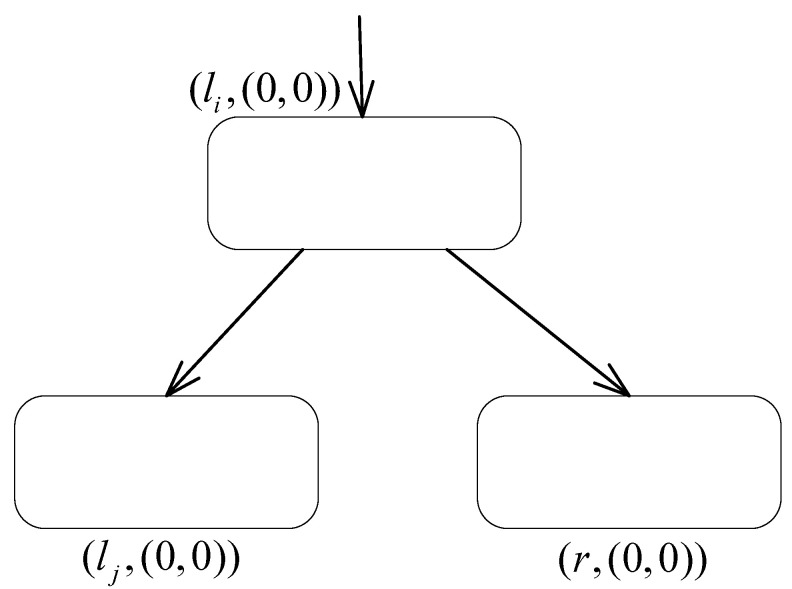
The deterministic ADD module of system $\Pi_{2}$.

**(2) The deterministic ADD module (Figure 8) to simulate a deterministic ADD instruction $l_{i}:\left(ADD\left(r\right),l_{j}\right)$.**

The computation process of the register machine $M_{2}$ in the accepting mode is deterministic and uses deterministic ADD instructions. Therefore, to achieve system simplification, the ADD module no longer applies inhibitory rules. Suppose that at step *t* neuron $\sigma_{l_{i}}$ receives one spike, and the system starts to simulate instruction $l_{i}:\left(ADD\left(r\right),l_{j}\right)$. by applying rule 0/a→a;0 to send one spike to neurons $\sigma_{l_{j}}$ and $\sigma_{r}$. Then, at step t+1, neuron $\sigma_{l_{j}}$ is activated, and the system starts to simulate instruction $l_{j}$ while the number of spikes contained in neuron $\sigma_{r}$ is added by one.

The SUB module, as shown in Figure 5, remains available for system $\Pi_{2}$. Meanwhile, in system $\Pi_{2}$, the FIN module is omitted, but the neuron $\sigma_{l_{h}}$ corresponding to the halt instruction is still retained to determine the acceptance result. The simulation of the system is terminated when one spike is received by neuron $\sigma_{l_{h}}$.

In summary, system $\Pi_{2}$ successfully simulates the register machine $M_{2}$ operating in the accepting mode. In total, three types of polarizations are applied in the system, and there are, at most, two rules contained in each neuron. Therefore, Theorem 2 is proven. □

Similarly, we present Table 2 to compare the resources of the MPAIRSN P systems with three SN P systems containing polarizations as number accepting devices. As can be seen, the MPAIRSN P system and the PASN P system use the least number of neurons.

## 4. A Small General MPAIRSN P System for Function Computation

This section constructs a small general MPAIRSN P system that performs function calculations.

**Theorem** **3.** *There is a Turing universal MPAIRSN P system using three types of polarizations with 95 neurons for computing functions.*

**Proof.**  Similarly, we prove the theorem by designing the MPAIRSN P system to simulate the register machine $M_{c}=\left(m,H,l_{0},l_{h},I\right)$. The machine serves to compute a function $f:N^{k}\to N$, in which, in the initial state, *k* specified registers are used to store *k* parameters (usually, the first *k* registers $r_{1},r_{2},\cdots r_{k}$ are selected), and the rest of the registers are empty. The machine operates from the start instruction $l_{0}$, followed by the execution of different instructions for processing, and the computation stops when it reaches the halt instruction $l_{h}$. At this point, the value of function *f* is stored in the specified register $r_{t}$, denoted by $M_{c}\left(n_{1},n_{2},\cdots ,n_{k}\right)$, where $n_{1},n_{2},\cdots ,n_{k}$ is *k* parameters. In addition, the machine used for the functions calculation works in a deterministic manner, so the ADD instruction form used is $l_{i}:\left(ADD\left(r\right),l_{j}\right)$. If it is assumed that $\left(\varphi_{0},\varphi_{1},\cdots \right)$ a fixed enumeration of unary partial recursive functions, for the machine $M_{c}$ to be universal as a function computing device, it is necessary that there exists a recursive function *g*, such that the equation $\varphi_{x}\left(y\right)=M_{c}\left(g\left(x\right),y\right)$ holds for all natural numbers *x*, *y*.

**Figure 9:**
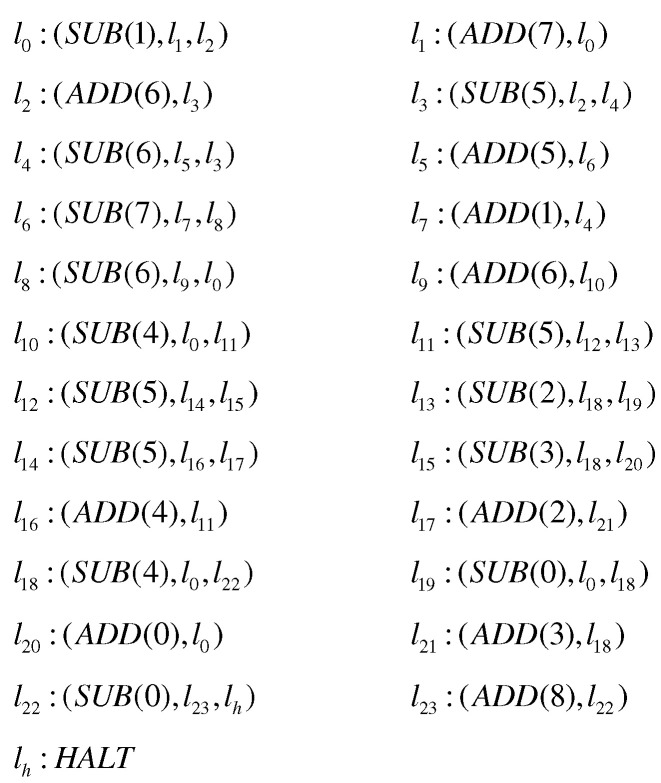
The universal register machine ${M^{\prime }}_{u}$.

According to the small universal register machine $M_{u}=\left(8,H,l_{0},l_{h},I\right)$ proposed by Korec [81], it contains eight registers (marked from 0 to 7) and 23 instructions. The machine firstly stores the parameters g(x) and *y* in registers 1 and 2, respectively, and the computation result $\varphi_{x}\left(y\right)$ is stored in register 0. Similarly, an MPAIRSN P system $\Pi_{3}$ is constructed to simulate the register machine $M_{u}$. To facilitate the system construction, the machine $M_{u}$ is modified by adding a new register 8 and replacing the original halt instruction with the instructions $l_{22}:\left(SUB\left(0\right),l_{23},l_{h}\right)$, $l_{23}:\left(ADD\left(8\right),l_{22}\right),l_{h}:HALT$. The modified register machine ${M^{\prime }}_{u}$ is shown in Figure 9, containing 24 ADD instructions and SUB instructions, 9 registers, and 25 labels.

**Figure 10:**
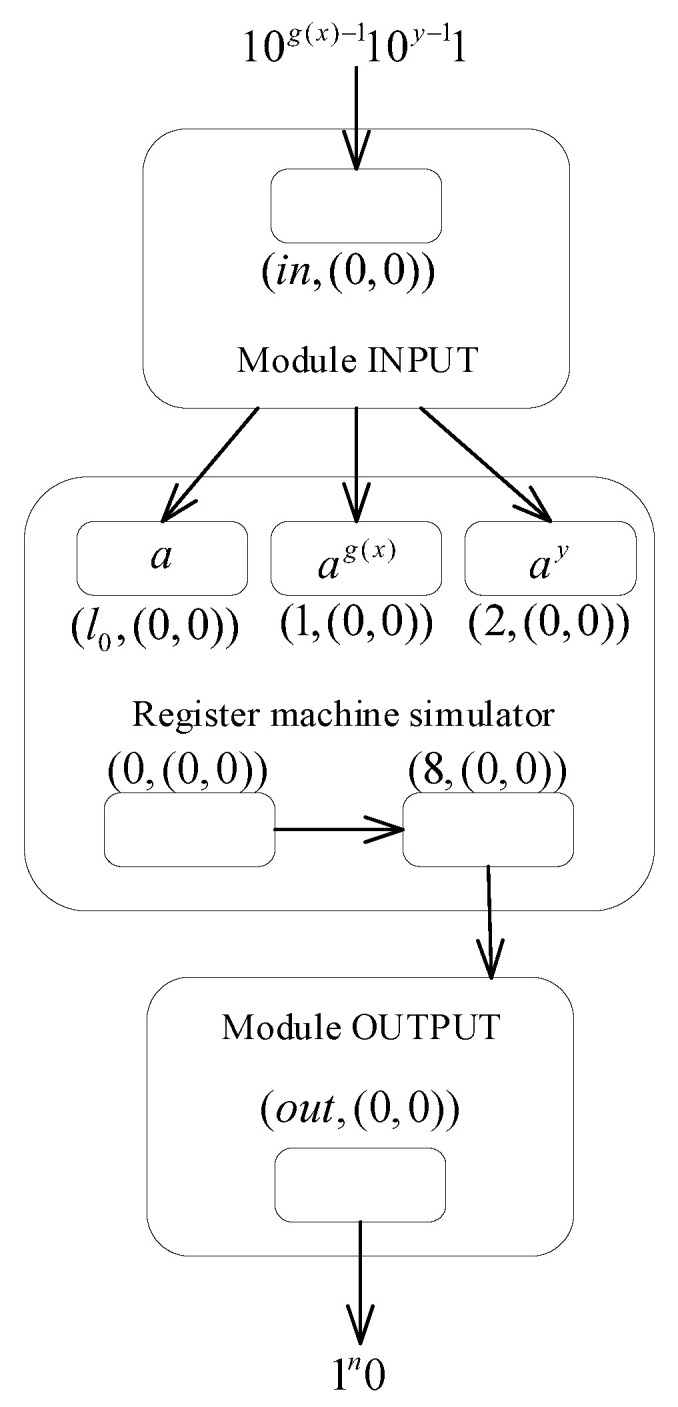
The overall construction of the MPAIRSN P system $\Pi_{3}$.

The overall composition of the small general MPAIRSN P system $\Pi_{3}$ (Figure 10) contains three main parts: the first is the INPUT module for reading the parameters encoded as spike sequences from the environment, the second is the simulation part of the register machine, which contains several deterministic ADD modules and SUB modules, and the third is the OUTPUT module for outputting the calculation results. The operation of the system begins with the parameters introduction phase, in which the neuron $\sigma_{in}$ in the INPUT module reads the spikes sequence. The spikes of amount g(x) and the spikes of amount *y* are introduced into neurons $\sigma_{1}$ and $\sigma_{2}$, respectively, as well as activating neuron $\sigma_{l_{0}}$. The system then proceeds to the register machine simulation phase. In this phase, a series of calculations are performed on the introduced parameters by simulating various instructions, and the final result is stored in neuron $\sigma_{8}$. Until neuron $\sigma_{l_{h}}$ is activated, the system calculation enters the result output phase. The neuron $\sigma_{out}$ outputs spikes to the environment in the OUTPUT module. We record the entire spikes received in the environment as the output result, corresponding to the value of the calculation result stored in register 8.

**Figure 11:**
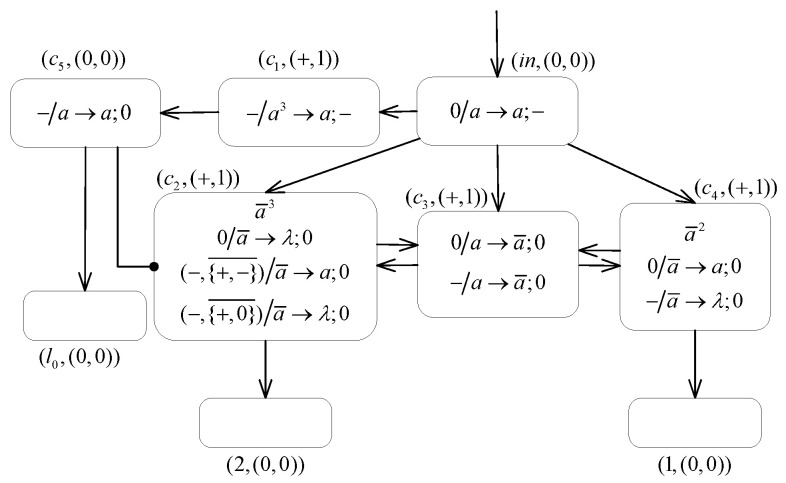
The INPUT module of system $\Pi_{3}$.

**(1) The INPUT module (Figure 11) for reading the spikes sequence $10^{g(x)}10^{y}1$ of encoded parameters from the environment.**

Suppose that at step *t*, neuron $\sigma_{in}$ receives the first spike in the sequence from the environment and fires. Then at step t+1, influenced by the firing of neuron $\sigma_{in}$, neurons $\sigma_{c_{2}}$, $\sigma_{c_{3}}$, and $\sigma_{c_{4}}$ all change to carry the neutral polarization. Thus, neurons $\sigma_{c_{3}}$ and $\sigma_{c_{4}}$ are available to trigger rules $0/a\to \bar{a};0\;$ and $0/\bar{a}\to a;0\;$, respectively, and the process of exchanging a spike and an anti-spike is continuously implemented in the subsequent steps. At the same time, one spike is continuously transmitted to neuron $\sigma_{1}$ at each step. However, for neuron $\sigma_{c_{2}}$, although it continuously receives spikes from neuron $\sigma_{c_{3}}$, all these spikes are deleted by the application of its internal forgetting rule $0/\bar{a}\to \lambda ;0$.

The above process proceeds until step t+g(x), neuron $\sigma_{in}$ receives the second spike in the spikes sequence and fires, and its firing produces one spike and a negative charge sent to neurons $\sigma_{c_{1}}$, $\sigma_{c_{2}}$, $\sigma_{c_{3}}$, and $\sigma_{c_{4}}$. Subsequently, the polarization states of neurons $\sigma_{c_{2}}$, $\sigma_{c_{3}}$, and $\sigma_{c_{4}}$ all turn negative. Then, the rule capable of triggering in neuron $\sigma_{c_{4}}$ is the forgetting rule $-/\bar{a}\to \lambda ;0$, and it is no longer sending spikes to neuron $\sigma_{1}$. At this point, the number of stored spikes in neuron $\sigma_{1}$ is exactly *g*(*x*). Additionally, the neuron $\sigma_{c_{2}}$ satisfies the triggering condition of its internal inhibitory rule $\left(-,\bar{\left\{+,-\right\}}\right)/\bar{a}\to a;0\;$, i.e., the formula $PAR_{c_{2}}=-\wedge PAR_{c_{5}}\notin \left\{+,-\right\}$ holds. Meanwhile, the rule applied by neuron $\sigma_{c_{3}}$ with negative polarization is $-/a\to \bar{a};0$. Then, in the subsequent steps, the mutual replenishment of spikes and ani-spikes between neurons $\sigma_{c_{2}}$ and $\sigma_{c_{3}}$ is achieved, and one spike is sent from neuron $\sigma_{c_{2}}$ to neuron $\sigma_{2}$ at each step.

At step t+g(x)+y, the neuron $\sigma_{in}$ receives the last spike in the spikes sequence and fires. Then, the polarization state of neuron $\sigma_{c_{1}}$ and the number of spikes contained are sufficient for triggering the rule $-/a^{3}\to a;-$. At step t+g(x)+y+2, the polarization state of neuron $\sigma_{c_{5}}$ becomes negatively influenced by the firing of the neuron $\sigma_{c_{1}}$. Additionally, the received spike in neuron $\sigma_{c_{1}}$ is subsequently consumed by the application of rule −/a→a;0. In the subsequent computation steps, the spikes or anti-spikes contained in neurons $\sigma_{c_{2}}$, $\sigma_{c_{3}}$, and $\sigma_{c_{4}}$ are cleared by the application annihilation rule, the forgetting rule, and the inhibitory forgetting rule, respectively. The neuron $\sigma_{l_{0}}$ is activated at step t+g(x)+y+3, while the numbers of spikes stored in neurons $\sigma_{1}$ and $\sigma_{2}$ at this step are g(x) and *y*, respectively. The above procedure successfully simulates the introduction of parameters g(x) and *y* into registers 1 and 2, respectively, and starts the simulation of the system $\Pi_{3}$.

Similarly, the ADD module, as shown in Figure 7, is applied in system $\Pi_{3}$ to simulate the deterministic ADD instruction of the machine ${M^{\prime }}_{u}$. The SUB module, as shown in Figure 4, remains available for system $\Pi_{3}$ to simulate the SUB instruction. The proof process has been illustrated in detail in the previous section.

**Figure 12:**
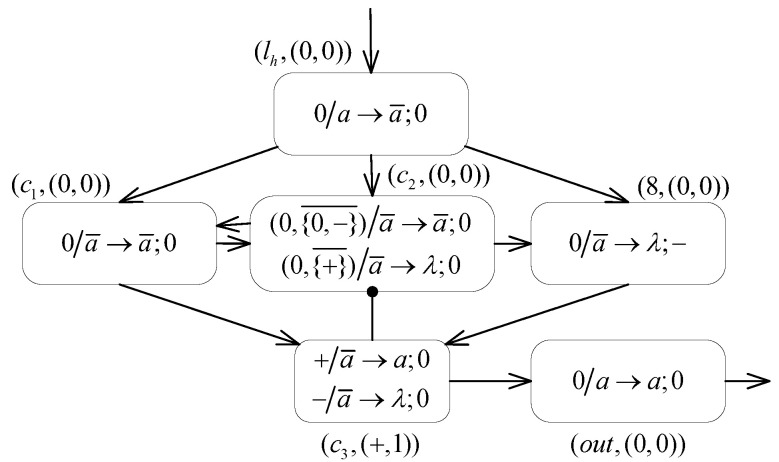
The OUTPUT module of system $\Pi_{3}$.

**(2) The OUTPUT module (Figure 12) outputs the result of the system calculation.**

Assuming that the neuron $\sigma_{l_{h}}$ is triggered and fires at step *t*, then at step t+1, after receiving an anti-spike from neuron $\sigma_{l_{h}}$, the neuron $\sigma_{8}$ consumes one spike by annihilation, where the number of spikes contained turns to n−1. Neurons $\sigma_{c_{1}}$ and $\sigma_{c_{2}}$ are allowed to trigger the spiking rule $0/\bar{a}\to \bar{a};0\;$ and the inhibitory spiking rule $\left(0,\bar{\left\{0,-\right\}}\right)/\bar{a}\to \bar{a};0$, respectively, thus enabling the two neurons to replenish spikes to each other and to fire at each step. One spike is sent by neuron $\sigma_{c_{2}}$ to neuron $\sigma_{8}$ at each step to successively annihilate its internal spikes. In addition, neuron $\sigma_{c_{3}}$ also receives an anti-spike from neuron $\sigma_{c_{1}}$ at each step, continuously applying rule $+/\bar{a}\to a;0\;$ to fire. Consequently, the neuron $\sigma_{c_{3}}$ feeds one spike to neuron $\sigma_{out}$ at each step, which subsequently triggers the rule 0/a→a;0 in neuron $\sigma_{out}$. The spikes generated by neuron $\sigma_{out}$ at each step are received in the environment, starting at step t+4.

The above process continues until step t+n, when the spikes contained in neuron $\sigma_{8}$ are completely annihilated and left empty, but one anti-spike from neuron $\sigma_{c_{2}}$ is still received. Subsequently, the neuron $\sigma_{8}$ applies rule $0/\bar{a}\to \lambda ;-\;$ to fire, and delivers a negative charge to neuron $\sigma_{c_{3}}$ to change its polarization. The neuron $\sigma_{c_{3}}$ only applies the forgetting rule $-/\bar{a}\to \lambda ;0\;$ after its polarization changes to negative without sending any spikes to the neuron $\sigma_{out}$. In addition, influenced by the changed polarization state of neuron $\sigma_{c_{3}}$, i.e., the formula $\alpha_{c_{2}}=0\wedge \alpha_{c_{3}}\notin \left\{+\right\}$ holds, only the inhibitory forgetting rule $\left(0,\bar{\left\{+\right\}}\right)/\bar{a}\to \lambda ;0\;$ is triggered in neuron $\sigma_{c_{2}}$, ensuring subsequent steps to clear neurons $\sigma_{c_{1}}$ and $\sigma_{c_{2}}$. At step t+n+3, the environment receives the last spike from neuron $\sigma_{out}$. The cumulative number of spikes received is (t+n+3)−(t+4)+1=n, which is exactly the number of spikes contained in neuron $\sigma_{8}$. That is, the module successfully outputs the computation result stored in register 8.

To summarize, the small universal proposed MPAIRSN P system $\Pi_{3}$ is capable of correctly simulating the universal register machine ${M^{\prime }}_{u}$ used for function computation. The required computational resources for this system are 98 neurons, which can be subdivided as shown in Table 3.

Moreover, observing the characteristics of the instructions of the universal register machine ${M^{\prime }}_{u}$ applied in Figure 9, the number of neurons needed for system $\Pi_{3}$ is reduced by integrating the SUB and ADD modules, which correspond to consecutive pairs of instructions.

**Figure 13:**
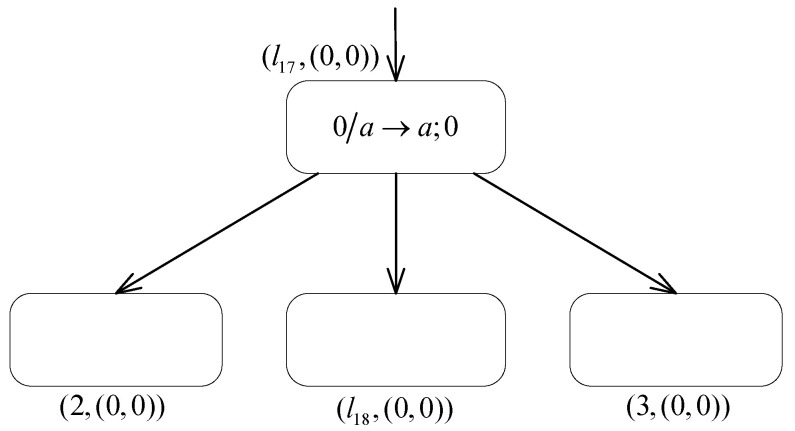
The ADD-ADD module of system $\Pi_{3}$.

**Figure 14:**
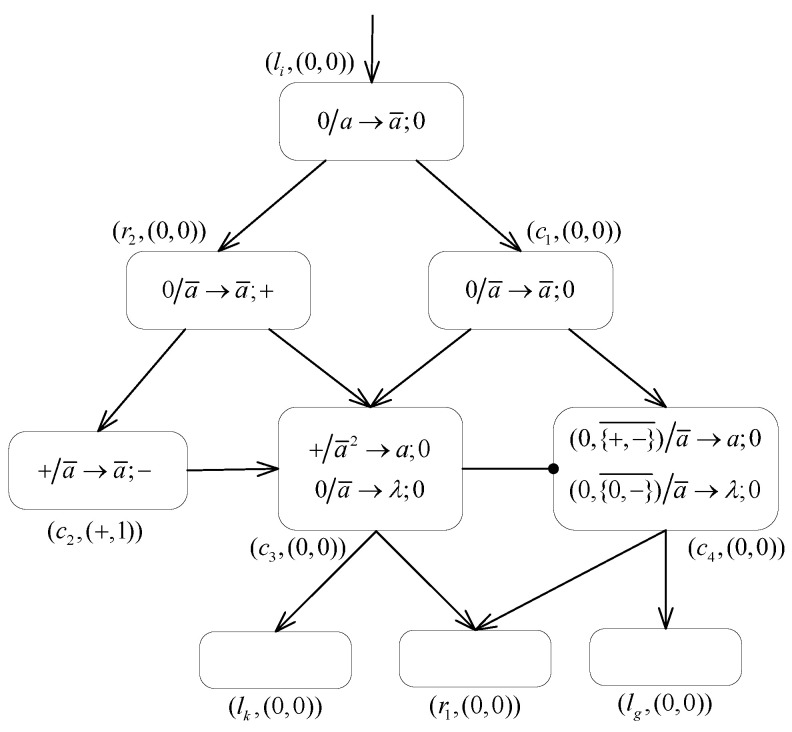
The ADD-SUB module of system $\Pi_{3}$.

(1) As for a pair of consecutive ADD instructions: $l_{17}:\left(ADD\left(2\right),l_{21}\right)$ and $l_{21}:\left(ADD\left(3\right),l_{18}\right)$, the ADD-ADD module, as shown in Figure 13, is constructed for simulation. In this way, the neuron $\sigma_{l_{21}}$ in the system is omitted.

(2) For two consecutive pairs of ADD-SUB instructions in ${M^{\prime }}_{u}$: $l_{5}:\left(ADD\left(5\right),l_{6}\right)$ and $l_{6}:\left(SUB\left(7\right),l_{7},l_{8}\right)$, $l_{9}:\left(ADD\left(6\right),l_{10}\right)$ and $l_{10}:\left(SUB\left(4\right),l_{0},l_{11}\right)$, both having the same form: $l_{i}:\left(ADD\left(r_{1}\right),l_{j}\right)$ and $l_{j}:\left(SUB\left(r_{2}\right),l_{g},l_{k}\right)$. For this purpose, the ADD-SUB module, as shown in Figure 14, allows the for merging of two modules. In this way, neurons $\sigma_{l_{6}}$ and $\sigma_{l_{10}}$ are omitted.

Thus, with the construction of the corresponding composite modules for consecutive pairs of instructions, the total number of neurons used by the system can be reduced by 3: one neuron is saved by the application of the ADD-ADD module, and two neurons are saved by the application of the ADD-SUB module. Thus, the system for function computation achieves universality by applying three types of polarizations and 95 neurons. In summary, Theorem 3 is proven. □

For the required computation resources and the computation speed, Table 4 shows the result of the proposed MPAIRSN P systems with four extended SN P systems, with polarizations for function computing. It should be noted that the computation resources here are the total number of neurons required for the systems to implement the function computation. The computation speed is the number of steps required by completing the computation. As is seen, compared to the remaining four SN P systems, the number of neurons used in the proposed MPAIRSN P system is still minimal. Moreover, the MPAIRSN P systems require the least number of steps to perform the computation, i.e., the computation is relatively faster.

## 5. Conclusions

In this paper, the concept of the membrane potential of nerve cells is introduced into the spiking neural P system, and the biological phenomena of charge accumulation and transmission between neurons are considered based on the spiking neural P systems with polarizations. Combining these ideas, a new variant called spiking neural P systems with membrane potential, inhibitory rules, and anti-spikes (MPAIRSN P systems for short) is proposed. The system adopts membrane potential as the rule-triggering condition and uses inhibitory rules to simulate the role of inhibitory synapses in the nervous system, which makes the construction and operation of the system more consistent with biological facts. The application of anti-spikes makes the system available with another information-encoding object, which simplifies the system construction and enhances the information representation capability of the system. The introduction of membrane potentials and the application of inhibitory rules provide the MPAIRSN P systems with more powerful control in computation.

We first give a formal definition of the MPAIRSN P system, and we update the novel inhibitory rule with membrane potential as the trigger condition. The conventions for neuronal charge calculation are given for determining neuron-associated polarization. By simplifying rules with polarization as the trigger condition, we design a small MPAIRSN P system as an illustrative example to detail its operation, which is equipped with the ability to generate arbitrary non-zero natural numbers. We demonstrate that MPAIRSN P systems are Turing-universal, as both a number generating and accepting device. A small universal MPAIRSN P system using 95 neurons is obtained, which saves 69 neurons compared to the initial spiking neural P systems with polarizations. By comparing the MPAIRSN P systems with other variants of the SN P systems with polarizations, it is shown that the MPAIRSN P systems have better performance, both in space efficiency and computation speed.

The MPAIRSN P system constructed in this paper mainly applies the standard spiking rules, and it is worth considering the use of extended rules in the system. It is also worthwhile to further investigate the computational power of the MPAIRSN P systems operating in other modes, such as sequential mode, asynchronous mode, etc. To reduce the number of polarizations and neurons needed, further research could consider introducing more biological features into the system without losing computational power, which allows the system to better approximate the biological facts. The spiking neural P systems with membrane potential, as a new variant, introduce a novel systematic rule-triggering mechanism, which is of novelty and development potential. It is worth considering the application of the spiking neural P systems with membrane potential to solve NP problems. Moreover, given that the MPAIRSN P systems provide powerful control over computation, it is practicable to apply them to real-world problems such as supervisory control and fault diagnosis.

## Figures and Tables

**Table 1:** Comparison of the extended SN P systems, with polarizations as number generating devices.

Computing Models	Configuration			
	Maximum Number of Rules per Neuron	Auxiliary Neurons (ADD)	Auxiliary Neurons (SUB)	Auxiliary Neurons (FIN)
MPAIRSN P systems	2	5	4	5
PSN P systems [71]	2	5	5	7
PASN P systems [72]	2	4	6	6
SNP–MCP systems [76]	2	4	6	1
PSNRS P systems [77]	2	7	8	5

**Table 2:** Comparison of the extended SN P systems with polarizations as number accepting devices.

Computing Models	Computation Resources		
	Maximum Number of Rules per Neuron	Auxiliary Neurons (ADD)	Auxiliary Neurons (INPUT)
MPAIRSN P systems	2	0	3
PASN P systems [72]	2	0	3
SNP–MCP systems [76]	2	2	5
PSNRS P systems [77]	2	2	7

**Table 3:** Computing resources required for the PAWSN P system $\Pi_{3}$.

Components of the PAWSN P System $\Pi_{3}$	Number of Neurons
Registers	9
Instruction labels	25
Auxiliary neurons required for SUB modules	56
Neurons required for INPUT modules	5
Neurons required for OUTPUT modules	3

**Table 4:** Comparison of the extended SN P systems with polarizations for computing functions.

Computing Models	Number of Neurons	Computation Speed
MPAIRSN P systems	95	*g*(*x*) + *y* + *n* + 58
PSN P systems [71]	164	*g*(*x*) + *y* + *n* + 128
PASN P systems [72]	121	*g*(*x*) + *y* + *n* + 72
SNP–MCP systems [76]	150	*g*(*x*) + *y* + *n* + 66
PSNRS P systems [77]	151	*g*(*x*) + *y* + *n* + 93

## Data Availability

No datasets were used in this article.
